# The experience of fertility concerns in patients with testicular cancer: a qualitative study

**DOI:** 10.1007/s00520-024-08720-y

**Published:** 2024-07-19

**Authors:** Ya Hu, Xue Fu, Xiaoya Jiang, Mengxiao Jiang, Xia Zheng, Huiming Lu, Man Xu

**Affiliations:** 1 Department of Urology, Sun Yat-Sen University Cancer Center, 651 Dongfeng Road East, Guangzhou, 510060 P.R. China; 2 State Key Laboratory of Oncology in Southern China, Guangzhou, 510060 P.R. China; 3 Guangdong Provincial Clinical Research Center for Cancer, Guangzhou, 510060 P.R. China; 4 International Medical Center, Zhujiang Hospital, Southern Medical University, Guangzhou, 510282 P.R. China

**Keywords:** Testicular cancer, Fertility concerns, Experience, Qualitative study

## Abstract

**Background:**

Testicular cancer usually occurs in young adult men between the ages of 20 and 40 years, which largely coincides with the age of men’s reproductive intentions. However, a serious side effect of testicular cancer therapy could reduce the fertility of patients.

**Purpose:**

To explore the experience of fertility concerns in patients with testicular cancer.

**Methods:**

A phenomenological research was conducted on 12 patients with testicular cancer. Data collection was from May 2023 to August 2023, and Colaizzi analysis method was used to analyze the data.

**Results:**

Four themes were found: (1) multiple worries and negative emotions, (2) fertility decision-making faces many challenges, (3) self-coping strategies for facing fertility concerns, (4) unmet supportive care needs.

**Conclusion:**

Medical staff should pay attention to the fertility needs of patients with testicular cancer and provide relevant interventions and support to reduce their fertility concerns.

## Introduction

Testicular cancer is a rare malignancy of the male urinary system, accounting for 1% of adult tumors and 5% of urologic tumors, with three to ten new cases per 100,000 men per year in Western countries [1]. Despite the increasing incidence of testicular cancer, it has a high cure rate with an overall long-term survival rate of 97% [2, 3]. Testicular cancer usually occurs in young adult men between the ages of 20 and 40 years [4]. The peak incidence of testicular cancer largely coincides with the age of men’s reproductive intentions [5]. However, cancerous lesions of the testes can impair their reproductive function, and previous study has shown that 6–24% of testicular cancer patients report azoospermia and 50% oligospermia at diagnosis [6]. It has been estimated that fertility is reduced by approximately 30% in patients with testicular cancer [7]. Meanwhile, the main treatment modalities for testicular cancer (e.g., surgery, chemotherapy, radiotherapy) could affect a patient’s sexual dysfunction, which can also cause some degree of damage to their reproductive function [8–10]. Therefore, the impact of disease and treatment on fertility has become the most important long-term sequelae of concern for testicular cancer patients of reproductive age [11].

Affected by the family planning policy, China’s fertility rate has been at a declining level for some time in the past [12], and even the opening of the two-child policy has not been able to change the predicament of the low fertility rate [13]. At the same time, the aging society will lead to a decline in the labor force, which will bring about a rise in the cost of labor and constrain the future development of the society and the economy, so that a continuous decline in the fertility rate has become a global problem.

Fertility concerns are individuals’ concerns about childbearing and child-rearing, including worries about reproductive capacity, their own health, their children’s health, and their children’s care [14]. Research indicated that the severity of reproductive concerns is positively associated with existential distress [15]. Moreover, reproductive concerns can result in negative perceptions of body image, loss of gender identity, and lower levels of hope and treatment adherence [16–18] which seriously affect patients’ long-term psychological well-being and quality of life [19].

Currently, studies used quantitative methods to explore the current status and influencing factors of fertility concerns in testicular cancer patients [16], but there is a lack of in-depth exploration of the real experience of fertility concerns and related needs of patients. Compared with quantitative research, qualitative research can provide a deeper and more profound understanding of patients’ disease experiences and feelings. Therefore, this study adopts a phenomenological qualitative research method to gain a deeper understanding of the experience of fertility concerns among testicular cancer patients and to analyze the existing problems, with a view to providing a basis for proposing and establishing a standardized fertility management process for testicular cancer patients.

## Methods

### Setting

From May 2023 to August 2023, the purposive sampling method was used to select young and middle-aged testicular cancer patients who received inpatient treatment in the Department of Urology, Sun Yat-Sen University Cancer Centre, P. R. China.

### Design and participants

The phenomenological research, as a kind of qualitative study, aims to describe individuals’ subjective encounters, employing inductive methods for comprehension and description to grasp genuine experiences [20]. Therefore, we conducted phenomenological research among young and middle-aged testicular cancer patients to understand their experience of fertility concerns. This study followed COREQ reporting guidelines [21]. Participants were recruited by an author who is a master-degree nurse using purposive sampling. The inclusion criteria were as follows: (1) aged 18 ~ 40 years old, (2) diagnosed with testicular cancer by pathologic examination, (3) patients with reproductive plans or reproductive needs, and (4) voluntarily participated in this study and gave informed consent. Exclusion criteria were as follows: (1) those with severe cognitive dysfunction or previous psychiatric history, (2) communication disorders. The sample size was determined by the construct of data saturation, that is the point at which no new information is obtained [22], which occurred at the twelfth interview.

### Data collection

Data were collected using semi-structured interviews with the young and middle-aged testicular cancer patients. All interviewees were interviewed after they had decided whether to freeze their sperm or not. The interview guide was developed by the research team according to the research aims and relevant literature (see Table 1). Two pilot interviews were conducted to identify unclear questions and revise the interview guide. After obtaining consent, the face-to-face interviews were conducted in a private and comfortable health education room. No other people were present besides the participants and researchers during the interview. Field notes were made during the interviews. No repeat interviews were carried out. A total of 12 interviews were conducted in this study, with each interview lasting from 20 to 45 min.

**Table 1:** Interview guide

1. Do you know how diseases and treatments affect fertility? In what ways did you learn about the effects of diseases and treatments on fertility?
2. How did you and your family feel when learned that the disease might affect fertility?
3. Do you know about fertility protection measures? How do you know about them?
4. What steps have you taken to cope with the effects of fertility concerns on you?
5. What support do you need most at the moment?

### Data analysis

The interview recordings were transcribed verbatim and uploaded to Nvivo software (version 11) for data management. Data analysis was performed in conjunction with data collection. The Colaizzi analysis method [20] was used to analyze the data, which encompasses a series of seven steps: (1) repeatedly reading through the transcripts and getting familiar with them and taking initial notes, (2) extracting meaningful statements that align with the studied phenomenon, (3) extrapolating and refining the meaning from meaningful statements, (4) searching for common concepts or characteristics of meaning and form themes, theme groups, and categories, (5) relating the topic to the research phenomenon for a complete description, (6) articulating the fundamental structure of the phenomenon, and (7) seeking feedback from participants to validate the authenticity of the findings.

In this manuscript, all quotations from participants are in italics. Aliases (N1 to 12) were used for the participants.

### Ethical considerations

This study was approved by the Sun Yat-Sen University Cancer Center ethics committee (IRB No: B2024-007–01). All participants provided written informed consent. The transcribed data were anonymized, and participants were informed that they would not be identifiable in any reports of this study. The participants could withdraw at any time without consequences. Only members of the research team had access to the audio recordings and transcripts.

### Trustworthiness

This study is conducted by researchers who have received systematic training and mastered qualitative research interview skills. Data collection, sorting, and analysis are carried out simultaneously. The study determined the sample size based on the fact that no new topics emerged at the time of the data analysis. Two researchers independently collated and analyzed the data and then compared the results until all the authors had reviewed the topic and reached a consensus. Two participants were invited to discuss the content and to ensure that the findings were clear and understandable.

## Results

### Characteristics of participants

Twelve interviewees were finally included in this study, whose general information is detailed in Table 2. A total of 12 interviews were conducted, the participants were aged 19–37 years.

**Table 2:** Characteristics of participants

No	Age (years)	Marital status at diagnosis	Number of children	Education	Frozen sperm (Y/N)	Treatment method
N1	28	Unmarried	0	Bachelor	Y	Surgery + chemo
N2	31	Married	0	Bachelor	Y	Surgery + chemo
N3	25	Married	1daughter	Junior college	Y	Surgery + chemo
N4	32	Married	0	Bachelor	Y	Surgery
N5	22	Unmarried	0	Bachelor	Y	Surgery
N6	29	Married	1 son	Senior college	N	Surgery + chemo
N7	33	Married	1 son and 1daughter	Bachelor	Y	Surgery + chemo
N8	27	Unmarried	0	Bachelor	N	Surgery
N9	21	Unmarried	0	Bachelor	N	Surgery
N10	37	Married	1 son and 1daughter	Senior college	N	Surgery
N11	19	Unmarried	0	Senior college	Y	Surgery
N12	32	Married	1 son	Bachelor	N	Surgery

### Themes and subthemes

**Fig. 1:**
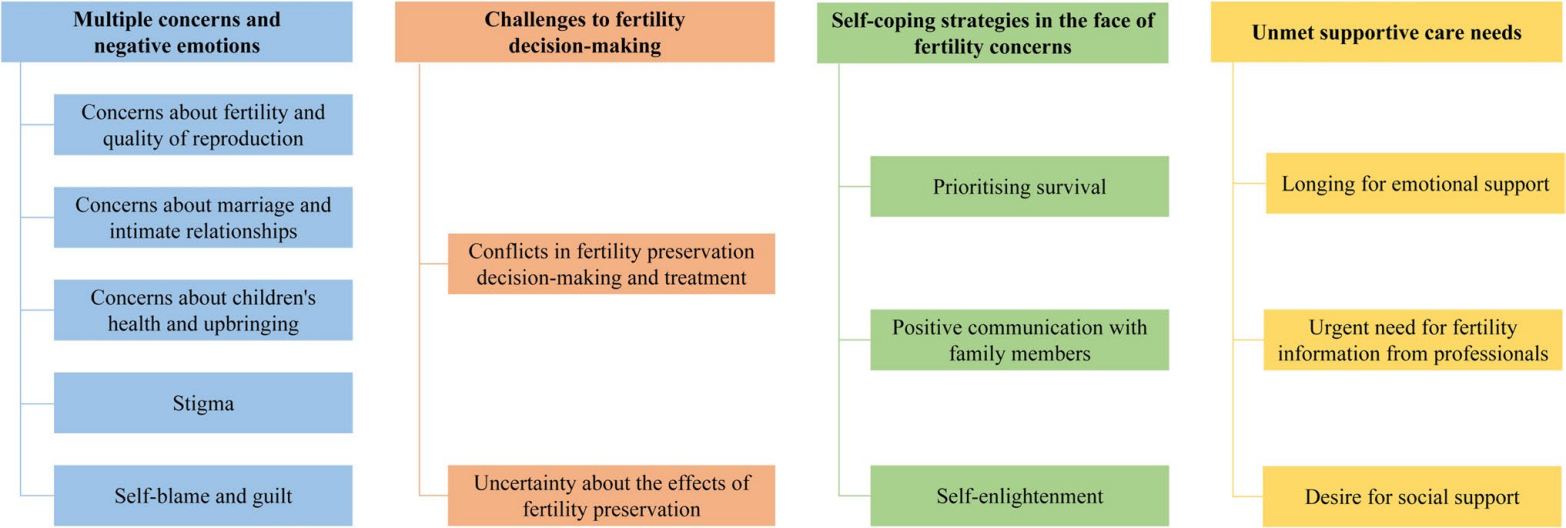
Themes and subthemes

Four themes and 13 subthemes were identified (Fig. 1):

1. Multiple concerns and negative emotions (concerns about fertility and quality of reproduction, concerns about marriage and intimate relationships, concerns about children’s health and upbringing, stigma, self-blame and guilt)
2. Challenges to fertility decision-making (conflicts in fertility preservation decision-making and treatment, uncertainty about the effects of fertility preservation)
3. Self-coping strategies in the face of fertility concerns (prioritizing survival, positive communication with family members; self-enlightenment)
4. Unmet supportive care needs (longing for emotional support, urgent need for fertility information from professionals, desire for social support).

#### Theme 1 multiple concerns and negative emotions

##### Concerns about fertility and quality of reproduction

Most of the testicular cancer patients have the desire to have children, but the tumor of the reproductive system makes them worry about their fertility and quality of reproduction, and they think that the disease of testicular cancer and the related chemotherapy have reduced their sperm activity and sperm quality, so as the number of sperms.

The doctor suggests that it will take one and a half years to prepare for pregnancy after chemotherapy, and we’ll be in trouble if we have a deformed child (N2).

What should I do if I have no fertility? Preparing for pregnancy depends on the genes, and it would be troublesome to give birth to a deformed child by then (N3).

When the ultrasound report came out saying it was cancer, I was more nervous because it involved the reproductive organs. Then I looked up on the internet that it is to be removed surgically, and I have no concept of the removal of this thing, so I think I will be more worried when I have no fertility after the removal (N5).

##### Concerns about marriage and intimate relationships

As both surgery and chemotherapy for testicular cancer will affect patients’ sexual function to different degrees, some patients will experience sexual dysfunction and worry about the impact on their married life afterwards, which will in turn lead to worries about the relationship between husband and wife.

There’s also the fact that the disease itself is quite embarrassing, and of course, I’m worried about how it will affect the life of husband and wife (N3).

There’s also a little bit of concern about physical needs, we are only in our 20 s (N6).

In addition, due to the uncertainty of their fertility, testicular cancer patients worry about their future romantic relationships, and they also worry about not knowing how and when to tell their partners about their illness in the future, which may cause the ending of the relationship, and then avoiding the possibility of a relationship.

It’s just that I don’t know when I should let my future partner know about the fact that I’m sick. I also don’t know if she will be able to accept this. I am worried that it will affect the continuity of the intimate relationship and that I will break up because of it (N5).

##### Concerns about children’s health and upbringing

Due to the inherent genetic risk of cancer, patients with testicular cancer are often concerned that their children may be at potential genetic risk, and they may believe that their sons are more susceptible. In addition, testicular cancer patients may also worry that their survival period is too short and the disease may recur and that they may not be able to take care of their children and see them grow up.

Artificial insemination I heard that you can choose the gender and the quality of the insemination, there is not much requirement for boys and girls, girls may be better because it may be passed on (to the male child). I’m worried about the possibility of having a male child(N4).

if I dead, my parents are almost 50 or 60 years old, they may not have the energy to raise my children and so will worry about my children’s upbringing (N6).

If I die years from now, what will happen to my children? Some people do have that fear. (N10).

##### Stigma

The diagnosis of testicular cancer may affect young and middle-aged men’s feelings about their masculinity, attractiveness, fertility, sexuality, and romantic relationships during the critical period of identity exploration, intimacy, and family formation [16]. In addition, comprehensive treatment modalities, mainly surgery and chemotherapy, can cause physical discomfort and changes in physical appearance, which can trigger body imagery disturbances and feelings of shame.

Only my family members know it. Of course, I hide it myself and don’t tell people that I have testicular cancer (N3).

I think not being able to have children for the rest of my life is no different from being disabled, I was ashamed of my inability to get an erection and infertility (N5).

##### Self-blame and guilt

Testicular cancer patients often harbored psychological burdens of guilt and self-blame towards the whole family, believing that they had failed to fulfil their family responsibilities and had brought financial burden and mental stress to their families.

My illness is a burden to my family, and they will be very worried about me(N10).

After I got sick, my family was always worried, I saw that they were in a bad mood and I might be affected too, so I was more depressed emotionally (N11).

#### Theme 2: challenges to fertility decision-making

##### Conflicts in fertility preservation decision-making and treatment 

Some patients require further chemotherapy after radical surgery for testicular cancer but miss out on sperm freezing due to the urgency of chemotherapy.

The doctor told me about a patient who had a biopsy taken during surgery and found lymph node metastasis, and then went straight to chemotherapy and didn’t have time to freeze his sperm (N1).

I had my sperm frozen when the quality wasn’t that good, but at that time, because of the imminent need for chemotherapy, I didn’t have the time to adjust my sperm quality until it’s good enough (before freezing the sperm) (N2).

##### Uncertainty about the effects of fertility preservation

Testicular cancer itself will affect the production of sperm, and the quality of sperm in patients will be lower than that of normal people [7], thus affecting the effectiveness of sperm freezing, and the effect of fertility preservation in patients is uncertain.

Even though I froze my sperm, I’m still worried. The doctor said that the probability of a normal person getting pregnant is 17%, and since my (sperm) concentration is only one-thirtieth of that of other people, it would be basically impossible to get pregnant, the probability is extremely low (N1).

When I had my sperm frozen, the quality of my sperm was not very good, so I was a little worried about whether the IVF would be successful or not; my sperm motility and quantity were not very good at that time, and I had to freeze my sperm twice to barely meet the criteria for freezing. I was still quite apprehensive during that time(N2).

#### Theme 3: self-coping strategies in the face of fertility concerns

##### Prioritizing survival 

Diagnosis and treatment of testicular cancer will bring direct harm to patients’ fertility, but most patients will still prioritize their survival before fertility preservation when making treatment-related decisions.

Fertility was not considered when choosing treatment options, or priority was given to treatment, and it is of no use to think too much about it, what is there to talk about fertility when your life is gone? (N1)

At that time, the doctor suggested that if we wanted to have children, we could delay chemotherapy, but my lover and I, still choose to be treated as early as possible, after all, life comes first, so we both put treatment as a priority(N2).

##### Positive communication with family members

Cancer patients share their disease-related emotions and thoughts with their spouses, which not only can help their spouses understand the changes in their emotional and physical status in time but also helps to enhance the tacit understanding between their spouses and themselves in coping with the challenges of the disease. Interviewees in the interviews indicated that after learning about the impact of the disease on fertility, they were more inclined to tell their spouses the facts in time and actively look for countermeasures.

I talked to my wife about the fertility aspect, and we both felt that we didn’t need to worry too much about it, and we have a child at the moment, so it would be better if we can (get pregnant) in the future, and it doesn’t matter if we really can’t (N6).

From the beginning I did talk to my girlfriend about the effect of the disease on fertility, and have not been hiding anything, and she is okay with it, we are both okay with infertility as an outcome(N8).

At the beginning my wife was more worried than I, and it was me who enlightened her later(N10).

In addition, the interviews indicated that they would also take the initiative to provide counseling and comfort to parents to reduce their tension and anxiety.

My parents are more worried about my fertility than I. My mum will search the internet herself if she doesn’t understand something. When she sees all the false information on the Internet, she will be even more scared, so I will try to comfort her and convince her that it’s okay (N3).

##### Self-enlightenment

After being told about the possible effects of the disease on fertility, testicular cancer patients, while experiencing apprehension and negative emotions, would also show a certain degree of psychological resilience and enlighten themselves so as to alleviate their inner worries.

I like to chat with my dad, and also play ball games, sing songs and so on, so that I can divert my attention, and I won’t intentionally think about this fertility problem and give myself mental comfort and tell myself it’s okay (N3).

Although having a tumor will affect my fertility, as I slowly learn about my condition, and the doctor said that mine is in the early stage, and that it was found and treated earlier, which was a blessing among misfortunes, so I have to be positive about it and treat it bravely anyway (N10).

In fact, there are still some positive cases in our favor, such as online reports that some athletes also cut off one side (testicles), but he still can be a very good athlete (N12).

#### Theme 4: unmet supportive care needs

##### Longing for emotional support

Some of the interviewees expressed a longing for family companionship and emotional support when faced with care needs arising from surgical treatments and worries about fertility issues.

If you don’t have the companionship and support of your family when you’re sick, who’s going to take care of and support you, I’m so lucky that my brother took care of me after my surgery, otherwise I wouldn’t have known what to do (N1)

Support is definitely needed, especially psychological support from family members, such as support from their closest wives(N10).’

##### Urgent need for fertility information support from professionals 

After a clear diagnosis of the disease, patients usually need to make decisions about treatment and fertility preservation, so most of the patients expressed the hope that they could get timely and professional knowledge about the disease and fertility from the medical staff, so as to facilitate the decision-making process of fertility preservation.

Sometimes patients really need timely advice from their doctors because sometimes they don’t know what the consequences of what they are doing are (N1).

I think healthcare professionals should give us guidance on fertility when patients are diagnosed, before surgery, and before chemotherapy, because at this point in time, I’m nearing the end of my treatment, so I hope that the healthcare professionals can tell me about this disease and what to expect in the future with some real-life examples(N3).

If possible, it would be better for the doctor to explain fertility related issues in advance, such as the suitable time and place for sperm freezing, etc., and what I should do if I decide to have a child later on(N4).

##### Desire for social support 

Because the site of the disease is a relatively hidden reproductive organ, and the disease and related treatments will affect the reproductive ability of the patient to varying degrees, some of the interviewees said that under the influence of traditional culture, the fact that they are unable to have children may not be accepted and understood by the society, which results in a serious psychological burden.

Both my parents are from the countryside, and they are very afraid of the people around them. discussions and different eyes, thinking that everyone else’s family has children but ours doesn’t, so the perceptions and views of people around me also affect me(N2).

I think part of the society still has a big prejudice against infertile people, for example, if a person is in his 40s or 50s and doesn’t have a child yet, people will say that you are infertile or something else(N5).

## Discussion

Fertility concerns in patients with testicular cancer can persist throughout the diagnosis of the disease, chemotherapy, and postoperative rehabilitation. The results of the present study showed the presence of not only fertility concerns, but also concerns about the health and parenting of their children and concerns about intimate relationships. Furthermore, the results also showed that testicular cancer patients, under the double blow of cancer and reduced fertility, usually have negative emotional experiences such as disease shame, guilt, and self-blame towards family members, which, if persisted for a long period of time, can lead to the emergence of adverse health outcomes and reduce their quality of life. Studies have shown that 28% of testicular cancer patients have high levels of fertility concern, with concerns about their own fertility ability being the most prominent [16]. Whereas fear of infertility or knowing that one’s fertility is compromised has a negative impact on the identity, masculinity, and mental health status of male cancer patients, and indirectly on their romantic and couple relationships. Therefore, we should pay attention to the subjective negative emotional experiences of testicular cancer patients, pay attention to and evaluate their psychological status, implement scientific psychological intervention programs, and encourage them to cope positively. At the same time, medical personnel should understand and strengthen the assessment of patients’ family social support system, encourage family members to strengthen communication with patients, and alleviate patients’ guilt towards their parents and partners due to fertility problems.

Previous study suggested [23] that fertility preservation is beneficial to alleviate cancer patients’ anxiety about future infertility. However, this study shows that patients often miss out on fertility preservation opportunities due to a lack of relevant information and facing conflicts with treatment decisions. A study that evaluated video messages related to testicular cancer on online platforms found that the content of the messages underestimated the impact of testicular cancer on fertility, which may mislead patients [24]. It is evident that testicular cancer patients lack access to reliable information. Therefore, correct preoperative consultation from the medical staff is necessary to provide the patient with reliable information. Moreover, studies have shown that the rate of sperm preservation in patients with testicular cancer is low [25], which is related to the lack of attention and dissemination of information about fertility preservation among healthcare professionals [26]. In addition, due to the urgency of treatment, some testicular cancer patients are often forced to give up fertility preservation due to the need for treatment. Therefore, after the diagnosis of cancer, healthcare professionals need to understand the fertility needs of patients and communicate with patients and their families as early as possible about fertility issues, so as to give patients and their families enough time for decision-making. European Association of Urology (EAU) also suggested that [8] healthcare professionals should fully inform patients of the fertility risk before treatment and inform patients of the measure of cryopreservation of spermatozoa as much as possible before orchiectomy, avoiding regrets of failing to take timely fertility preservation measures in the future.

For testicular cancer patients, good psychological resilience can help them reduce anxiety and depression, enhance their confidence in the face of disease, and improve the quality of survival [27]. The statements of some patients in this study indicate that they have a certain level of psychological resilience; however, studies have shown that the level of psychological resilience of cancer patients is a dynamic process of change [28], so how to maintain and improve the level of psychological resilience of patients with testicular cancer is an issue that should be paid attention to by medical personnel. There are a variety of measures that have been shown to be effective in improving patients’ mental resilience; for instance, medical personnel should pay dynamic attention to the abnormal psychological changes of patients in all stages of treatment and give timely intervention and treatment; secondly, multiple parties can be involved in building a good online social media platform for patients [29], to promote the maintenance of patients’ social interactions and interpersonal relationships, and better improve the patients’ mental health [30, 31]. In addition, it has been shown that positive thoughts provide individuals with self-regulatory energy to better cope with negative events in their lives [32]. Therefore, when patients have anxiety, depression, or large mood fluctuations, psychotherapists can intervene to provide psychological counseling to patients and encourage patients to master psychological adjustment skills such as positive thinking, so as to enable patients to deal with the fertility concerns more positively.

In addition, some patients may have fertility concerns due to social pressures. In this study, some of the interviewees expressed that because of the inability to have children normally, they and their parents would be viewed differently by people around them, and there is a certain degree of psychological pressure. Fertility issues are also inevitably talked about as a social topic in daily life, so infertile people have to deal with not only the pressures of reproductive health problems, but also the pressures of social opinion. A good social support system has a positive effect on the psychological pressure on the patients [33], so it is very important to correctly understand the problem of reproduction, to create a harmonious social atmosphere, and to strengthen the construction of the social support system for the patients with testicular cancer. Firstly, the emotional support from spouses and parents should be strengthened to create a harmonious and beautiful family atmosphere, so as to alleviate the patients’ worries and guilt due to the impact of the disease on fertility; secondly, the government and the relevant departments should publicize and promote the correct values and concepts of fertility and avoid being influenced by traditional concepts such as “passing on the family lines” and looking at the issue of reproduction with unpleasant and discriminatory attitudes and should be tolerance and understanding. Through these different means, we can establish a solid social support system for testicular cancer patients, and then effectively alleviate the fertility concerns of them.

## Limitations

The limitation of this study is that it only explores the experience of fertility concerns from the patients’ perspective and lacks the perspective of the patients’ spouses and family members. It is necessary to pay attention to the experience of the patients’ spouses and parents, to gain a more in-depth and comprehensive understanding of the factors that affect the fertility concerns of the patients with testicular cancer from different perspectives, and to provide the basis for formulating effective intervention strategies to reduce the level of reproductive anxiety in testicular cancer.

## Conclusion

This study describes the experience of fertility concerns faced by patients with testicular cancer and finds that patients with testicular cancer not only have multiple worries and related negative emotions, but also face many challenges in the process of fertility decision-making, as well as the actual existence of multiple supportive care needs. It is suggested that healthcare professionals should pay attention to the fertility problems of testicular cancer patients and provide diversified psychological interventions, information support, and emotional support to meet the fertility-related information needs of patients and their families.

## Data Availability

No datasets were generated or analyzed during the current study.

## References

[1] Park JS, Kim J, Elghiaty A et al (2018) Recent global trends in testicular cancer incidence and mortality. Medicine 97(37). 10.1097/md.0000000000012390 10.1097/MD.0000000000012390 PMC6155960 30213007

[2] Nigam M, Aschebrook-Kilfoy B, Shikanov S, Eggener S (2015) Increasing incidence of testicular cancer in the United States and Europe between 1992 and 2009. World J Urol 33(5):623–631. 10.1007/s00345-014-1361-y 25030752 10.1007/s00345-014-1361-y

[3] Gurney JK, Florio AA, Znaor A, Ferlay J, Laversanne M, Sarfati D, Bray F, McGlynn KA (2019) International trends in the incidence of testicular cancer: lessons from 35 years and 41 countries. Eur Urol 76(5):615–623. 10.1016/j.eururo.2019.07.002 31324498 10.1016/j.eururo.2019.07.002 PMC8653517

[4] Loveday C, Law P, Litchfield K, Levy M, Holroyd A, Broderick P, Kote-Jarai Z, Dunning AM, Muir K, Peto J, Eeles R, Easton DF, Dudakia D, Orr N, Pashayan N, Reid A, Huddart RA, Houlston RS, Turnbull C (2018) Large-scale analysis demonstrates familial testicular cancer to have polygenic aetiology. Eur Urol 74(3):248–252. 10.1016/j.eururo.2018.05.036 29935977 10.1016/j.eururo.2018.05.036

[5] Parekh NV, Lundy SD, Vij SC (2020) Fertility considerations in men with testicular cancer. Transl Androl Urol 9(Suppl 1):S14-s23. 10.21037/tau.2019.08.08 32055481 10.21037/tau.2019.08.08 PMC6995852

[6] Moody JA, Ahmed K, Yap T, Minhas S, Shabbir M (2019) Fertility managment in testicular cancer: the need to establish a standardized and evidence-based patient-centric pathway. BJU Int 123(1):160–172. 10.1111/bju.14455 29920910 10.1111/bju.14455

[7] Huyghe E, Matsuda T, Daudin M, Chevreau C, Bachaud JM, Plante P, Bujan L, Thonneau P (2004) Fertility after testicular cancer treatments: results of a large multicenter study. Cancer 100(4):732–737. 10.1002/cncr.11950 14770428 10.1002/cncr.11950

[8] Patrikidou A, Cazzaniga W, Berney D, Boormans J, de Angst I, Di Nardo D, Fankhauser C, Fischer S, Gravina C, Gremmels H, Heidenreich A, Janisch F, Leão R, Nicolai N, Oing C, Oldenburg J, Shepherd R, Tandstad T, Nicol D (2023) European Association of Urology Guidelines on testicular cancer: 2023 update. Eur Urol 84(3):289–301. 10.1016/j.eururo.2023.04.010 37183161 10.1016/j.eururo.2023.04.010

[9] Bandak M, Jørgensen N, Juul A, Lauritsen J, Gundgaard Kier MG, Mortensen MS, Daugaard G (2017) Preorchiectomy Leydig cell dysfunction in patients with testicular cancer. Clin Genitourin Cancer 15(1):e37–e43. 10.1016/j.clgc.2016.07.006 27524512 10.1016/j.clgc.2016.07.006

[10] Raffo M, Di Naro A, Napolitano LA-O, Aveta AA-O, Cilio SA-O, Pandolfo SA-O, Manfredi CA-O, Lonati CA-O, Suardi NR (2024) Testicular cancer treatments and sexuality: a narrative review. Medicina 60(4):586. 10.3390/medicina60040586 38674232 10.3390/medicina60040586 PMC11051825

[11] Hamano I, Hatakeyama S, Ohyama C (2017) Fertility preservation of patients with testicular cancer. Reprod Medicine Biology 16(3):240–251. 10.1002/rmb2.12037 10.1002/rmb2.12037 PMC5715882 29259474

[12] Jiang Q, Li Y, Sanchez-Barricarte JJ (2016) Fertility intention, son preference, and second childbirth: survey findings from Shaanxi Province of China. Soc Indic Res 125(3):935–953. 10.1007/s11205-015-0875-z 28769144 10.1007/s11205-015-0875-z PMC5536174

[13] Yang Y, He R, Zhang N et al (2023) Second-child fertility intentions among urban women in China: a systematic review and meta-analysis. Int J Envr Res Public Health 20 (4). 10.3390/ijerph20043744 10.3390/ijerph20043744 PMC9962327 36834437

[14] Gorman JR, Su HI, Pierce JP, Roberts SC, Dominick SA, Malcarne VL (2014) A multidimensional scale to measure the reproductive concerns of young adult female cancer survivors. J Cancer Surviv: Res Pract 8(2):218–228. 10.1007/s11764-013-0333-3 10.1007/s11764-013-0333-3 PMC4016119 24352870

[15] Carr AL, Roberts S, Bonnell LN et al (2022) Existential distress and meaning making among female breast cancer patients with cancer-related fertility concerns. Pall Supp Care 1–9. 10.1017/s1478951522001675 10.1017/S1478951522001675 36562084

[16] Ljungman L, Eriksson LE, Flynn KE, Gorman JR, Ståhl O, Weinfurt K, Wiklander M, Lampic C, Wettergren L (2019) Sexual dysfunction and reproductive concerns in young men diagnosed with testicular cancer: an observational study. J Sex Med 16(7):1049–1059. 10.1016/j.jsxm.2019.05.005 31255211 10.1016/j.jsxm.2019.05.005

[17] Wang Y, Logan S, Stern K, Wakefield CE, Cohn RJ, Agresta F, Jayasinghe Y, Deans R, Segelov E, McLachlan RI, Gerstl B, Sullivan E, Ledger WE, Anazodo A (2020) Supportive oncofertility care, psychological health and reproductive concerns: a qualitative study. Support Care Cancer: Off J Multinat Assoc Support Care Cancer 28(2):809–817. 10.1007/s00520-019-04883-1 10.1007/s00520-019-04883-1 31154532

[18] Xie J, Sun Q, Duan Y, Cheng Q, Luo X, Zhou Y, Liu X, Xiao P, Cheng ASK (2022) Reproductive concerns among adolescent and young adult cancer survivors: a scoping review of current research situations. Cancer Med 11(18):3508–3517. 10.1002/cam4.4708 35332694 10.1002/cam4.4708 PMC9487873

[19] Benedict C, Thom B, Friedman DN, Pottenger E, Raghunathan N, Kelvin JF (2018) Fertility information needs and concerns post-treatment contribute to lowered quality of life among young adult female cancer survivors. Support Care Cancer: Off J Multinat Assoc Support Care Cancer 26(7):2209–2215. 10.1007/s00520-017-4006-z 10.1007/s00520-017-4006-z PMC5984121 29387996

[20] Li Z, Liu Y (2019) Nursing research methods [M], 2nd edn. People’s Medical Publishing House, Beijing, p 21

[21] Tong A, Sainsbury P, Craig J (2007) Consolidated criteria for reporting qualitative research (COREQ): a 32-item checklist for interviews and focus groups. Int J Qual Health Care 19:349 17872937 10.1093/intqhc/mzm042

[22] Moser A, Korstjens I (2018) Series: practical guidance to qualitative research. Part 3: sampling, data collection and analysis. Eur J Gen Pract 24(1):9–18. 10.1080/13814788.2017.1375091 29199486 10.1080/13814788.2017.1375091 PMC5774281

[23] Armuand GM, Wettergren L, Rodriguez-Wallberg KA, Lampic C (2015) Women more vulnerable than men when facing risk for treatment-induced infertility: a qualitative study of young adults newly diagnosed with cancer. Acta oncologica (Stockholm, Sweden) 54(2):243–252. 10.3109/0284186x.2014.948573 25140859 10.3109/0284186x.2014.948573

[24] Di Bello F, Collà Ruvolo CA-O, Cilio S, La Rocca R, Capece M, Creta MA-O, Celentano G, Califano G, Morra S, Iacovazzo C, Coviello A, Buonanno P, Fusco F, Imbimbo C, Mirone V, Longo N (2022) Testicular cancer and youtube: what do you expect from a social media platform? Int J Urol 29(7). 10.1111/iju.14871 10.1111/iju.14871 35318754

[25] Sonnenburg DW, Brames MJ, Case-Eads S, Einhorn LH (2015) Utilization of sperm banking and barriers to its use in testicular cancer patients. Support Care Cancer: Off J Multinat Assoc Support Care Cancer 23(9):2763–2768. 10.1007/s00520-015-2641-9 10.1007/s00520-015-2641-9 25680764

[26] Stark SS, Natarajan L, Chingos D, Ehren J, Gorman JR, Krychman M, Kwan B, Mao JJ, Myers E, Walpole T, Pierce JP, Su HI (2019) Design of a randomized controlled trial on the efficacy of a reproductive health survivorship care plan in young breast cancer survivors. Contemp Clin Trials 77:27–36. 10.1016/j.cct.2018.12.002 30553078 10.1016/j.cct.2018.12.002 PMC6754982

[27] Adamkovič M, Fedáková D, Kentoš M, Bozogáňová M, Havrillová D, Baník G, Dědová M, Piterová I (2022) Relationships between satisfaction with life, posttraumatic growth, coping strategies, and resilience in cancer survivors: a network analysis approach. Psychooncology 31(11):1913–1921. 10.1002/pon.5948 35524705 10.1002/pon.5948 PMC9790334

[28] Sihvola S, Kuosmanen L, Kvist T (2022) Resilience and related factors in colorectal cancer patients: a systematic review. Eur J Oncol Nurs: Off J Eur Oncol Nurs Soc 56:102079. 10.1016/j.ejon.2021.102079 10.1016/j.ejon.2021.102079 34844135

[29] Wang Y, Bao S, Chen Y (2023) How does social media use influence the mental health of pancreatic cancer patients: a chain mediating effect of online social support and psychological resilience. Front Public Health 11. 10.3389/fpubh.2023.1166776 10.3389/fpubh.2023.1166776 PMC10333754 37441643

[30] Kim YA-O, Fingerman KL (2022) Daily social media use, social ties, and emotional well-being in later life. J Soc Pers Relat 39(6):1794–1813. 10.1177/02654075211067254 37727534 10.1177/02654075211067254 PMC10508904

[31] O’Reilly M, Dogra N, Hughes J, Reilly P, George R, Whiteman N (2018) Potential of social media in promoting mental health in adolescents. Health Promot Int 34(5):981–991. 10.1093/heapro/day056 10.1093/heapro/day056 PMC6904320 30060043

[32] Stephen AE, Mehta DH (2019) Mindfulness in surgery. Am J Lifestyle Med 13(6):552–555. 10.1177/1559827619870474 31662720 10.1177/1559827619870474 PMC6796221

[33] Garg R, Rebić N, De Vera MA (2020) Information needs about cancer treatment, fertility, and pregnancy: qualitative descriptive study of Reddit threads. JMIR Cancer 6(2):e17771. 10.2196/17771 33263547 10.2196/17771 PMC7744261

